# Protection of TGF-β1 against Neuroinflammation and Neurodegeneration in Aβ_1–42_-Induced Alzheimer’s Disease Model Rats

**DOI:** 10.1371/journal.pone.0116549

**Published:** 2015-02-06

**Authors:** Jia-Hui Chen, Kai-Fu Ke, Jian-Hua Lu, Yi-Hua Qiu, Yu-Ping Peng

**Affiliations:** 1 Department of Physiology, School of Medicine, and Co-innovation Center of Neuroregeneration, Nantong University, Nantong, China; 2 Department of Neurology, Affiliated Hospital, Nantong University, Nantong, China; Indiana School of Medicine, UNITED STATES

## Abstract

Neuroinflammation has been reported to be associated with Alzheimer’s disease (AD) pathogenesis. Neuroinflammation is generally considered as an outcome of glial activation; however, we recently demonstrated that T helper (Th)17 cells, a subpopulation of proinflammatory CD4+ T cells, are also involved in AD pathogenesis. Transforming growth factor (TGF)-β1, a cytokine that can be expressed in the brain, can be immunosuppressive, but its effects on lymphocyte-mediated neuroinflammation in AD pathogenesis have not been well addressed. In the current study we administered TGF-β1 via intracerebroventricle (ICV) and intranasal (IN) routes in AD model rats to investigate its antiinflammatory and neuroprotective effects. The AD rat model was prepared by bilateral hippocampal injection of amyloid-β (Aβ)_1–42_. TGF-β1 was administered via ICV one hour prior to Aβ_1–42_ injection or via both nares seven days after Aβ_1–42_ injection. ICV administration of TGF-β1 before Aβ_1–42_ injection remarkably ameliorated Aβ_1–42_-induced neurodegeneration and prevented Aβ_1–42_-induced increases in glia-derived proinflammatory mediators (TNF-α, IL-1β and iNOS), as well as T cell-derived proinflammatory cytokines (IFN-γ, IL-2, IL-17 and IL-22), in the hypothalamus, serum or cerebrospinal fluid (CSF) in a concentration-dependent manner. TGF-β1 pretreatment also prevented Aβ_1–42_-induced decreases in the neurotrophic factors, IGF-1, GDNF and BDNF, and in the antiinflammatory cytokine, IL-10. Similarly, IN administration of TGF-β1 after Aβ_1–42_ injection reduced neurodegeneration, elevation of proinflammatory mediators and cytokines, and reduction of neurotrophic and antiinflammatory factors, in the hypothalamus, serum or CSF. These findings suggest that TGF-β1 suppresses glial and T cell-mediated neuroinflammation and thereby alleviates AD-related neurodegeneration. The effectiveness of IN administered TGF-β1 in reducing Aβ_1–42_ neurotoxicity suggests a possible therapeutic approach in patients with AD.

## Introduction

Neuroinflammation has been widely recognized as a possible pathological contributor to Alzheimer’s disease (AD), an age-dependent neurodegenerative disorder and the most common cause of dementia [1, 2]. Neuroinflammation is generally considered to result from activation of glial cells including microglia and astrocytes [3], and activated microglia are detected in AD patient brains [4]. These activated microglia release cytotoxic compounds, cytokines and chemokines, which are believed to eventually cause neuronal damage and death [4]. Indeed, pharmacologic inhibition of microglial inflammatory responses confers neuroprotection [5, 6]. In addition to glial cells, recent studies have revealed that lymphocytes, especially T lymphocytes, participate in AD-associated neuroinflammation [7]. We have recently reported parenchymal infiltration of T helper (Th)17 cells, a subpopulation of proinflammatory CD4^+^ T cells, via a disrupted blood-brain barrier (BBB) and elevation of the Th17 proinflammatory cytokines, interleukin (IL)-17 and IL-22 in amyloid-β (Aβ)_1–42_-induced AD brain [8]. These findings suggest that peripheral Th17 cells can penetrate into the brain and mediate AD neuroinflammation. In addition to Th17 cells, the involvement of other subsets of CD4^+^ T cells including proinflammatory and antiinflammatory T cells, such as Th1, Th2 and regulatory T (Treg) cells, still remains to be clarified in the neuroinflammatory pathogenesis of AD.

Transforming growth factor (TGF)-β1, an immunosuppressive cytokine, is an important regulator of cell growth and differentiation and of tissue repair after injury [9, 10]. It also protects neurons against damage induced by excitotoxins, hypoxia/ischemia and trophic factor deprivation [11–14]. TGF-β1 has been shown to play a pivotal role in AD pathogenesis [15]. For example, TGF-β1 level in the plasma of AD patients is reduced [16], and TGF-β1 secretion from peripheral blood mononuclear cells in the circulation of AD patients is also decreased [17]. Importantly, overexpression of TGF-β1 prominently reduces plaque formation and Aβ accumulation in hAPP mice, and this effect of TGF-β1 is related to enhanced Aβ clearance by BV-2 microglia [18], suggesting that microglia are involved in the mechanism of TGF-β1 neuroprotection against Aβ aggregation-induced neurotoxicity. However, it is less clear whether TGF-β1 inhibits T cell-mediated neuroinflammation and alleviates AD-associated neurodegeneration when given exogenously. Clarifying this point is of clinical importance for developing therapeutic strategies to prevent and treat AD.

In most experimental systems showing neuroprotection, TGF-β1 is applied by intracerebroventricular (ICV) administration before brain injury. Although useful in demonstrating protection by TGF-β1, the preventive strategy is not practical in real patients. Intranasal (IN) administration is an effective and non-invasive approach for treatment with growth factors that bypasses the BBB to the brain along the olfactory and trigeminal neural pathways [19, 20]. IN administration of TGF-β1 reduces infarct volume, improves functional recovery and enhances neurogenesis in mice after stroke [21]. However, it is not known whether TGF-β1 administration via IN after Aβ_1–42_ toxicity can ameliorate AD-associated neuroinflammation or neurodegeneration. Accordingly, in addition to ICV administration of TGF-β1 prior to Aβ_1–42_ injection, IN administration of TGF-β1 after Aβ_1–42_ injection was also used in the present study to explore a more clinically relevant therapeutic approach for patients with AD.

Aβ is widely recognized as a pathogenic factor in AD that can be directly neurotoxic as well as provoke neuroinflammatory responses by activating microglia [22]. Intrahippocampal injection of Aβ_1–42_ in rat brain imitates several pathological and behavioral features of AD patients, including inflammatory reactivity present in human AD brain [23]. Thus, in this study, we employed Aβ_1–42_ by intrahippocampal injection in rats to induce AD-associated changes, including disruption of spatial learning and memory, amyloid precursor protein (APP) and protein phosphatase (PP)2A expression, neuronal loss and apoptosis, glial cell activation, and proinflammatory and antiinflammatory T cell responses.

## Materials and Methods

### Ethics Statement

The animal work performed in this study followed the National Institute of Health Guide for the Care and Use of Laboratory Animals and was approved by the Institutional Animal Care and Use Committee of Nantong University.

### Animals

Four-month old Sprague-Dawley rats (Center of Experimental Animals, Nantong University, China) were kept on a 12-hour light/dark cycle, and housed individually with free access to food and water. The rats were randomly divided into six groups for ICV administration of TGF-β1: intact, bilateral hippocampal saline injection, bilateral hippocampal Aβ_1–42_ injection, and TGF-β1 treatment with three concentrations, 4, 10 or 50 ng in 5 μl, respectively, via ICV administration before Aβ_1–42_ injection. For IN administration of IGF-β1, there were the four groups: intact, bilateral hippocampal saline injection, bilateral hippocampal Aβ_1–42_ injection, and TGF-β1 treatment via IN after Aβ_1–42_ injection. Intact and saline-treated animals were used as controls. There were 12 rats in each group, and therefore a total of 120 rats were used in the current study.

### Preparation of Aβ_1–42_-Induced AD Rat Model

Before injection, Aβ_1–42_ (Sigma-Aldrich, USA) was incubated in sterile saline at 37°C for 7 days to allow peptide assembly associated with toxicity [24]. The incubated Aβ_1–42_ solutions generally contain both fibril-like structures and different-sized oligomers [25]. Rats that had been deeply anesthetized with pentobarbital (55 mg/kg, i.p.) and mounted in a stereotactic frame (David Kopf 902-A, USA) were injected with the incubated Aβ_1–42_ solution into each side of the hippocampus with a volume of 1 μl containing 4 μg Aβ_1–42_ using the following stereotaxic coordinates: 3.6 mm posterior to bregma, 2.4 mm left/right to midline, and 2.8 mm ventral to bregma [26]. Each injection was performed over 5 min and following injection, the needle remained in the target location for 10 min to avoid Aβ_1–42_ reflux along the needle tract. After surgery, each rat was injected with penicillin (100,000 U) in the hindquarter muscle to prevent infection.

### TGF-β1 Treatment

There were two routes for TGF-β1 treatment, ICV administration before Aβ_1–42_ injection and IN administration after Aβ_1–42_ injection. For the ICV administration of TGF-β1, TGF-β1 (R&D Systems, Germany) was dissolved in sterile saline at concentrations of 0.8 ng/μl, 2 ng/μl, or 10 ng/μl. One hour prior to Aβ_1–42_ injection, 5 µl of TGF-β1 solution was injected into the left lateral cerebral ventricle with the following coordinates: 0.8 mm posterior to bregma, 1.5 mm lateral from the midline, and 3.8 mm ventral from the skull. Thus, three different concentrations of TGF-β1 were used via ICV administration: 4 ng, 10 ng and 50 ng in 5 μl. IN administration of TGF-β1 was performed as described by Liu et al. [27, 28] with modifications. On the 7th day after Aβ_1–42_ injection, rats were anesthetized with pentobarbital (55 mg/kg, i.p.) and placed on their backs. A total volume of 30 μl solution per rat containing 1.5 μg TGF-β1 was given as 2 μl drops into left and right nares, alternating sides at 2 min intervals over a period of 30 min. The mouth and opposite naris were shut during the administration. On day 7 following TGF-β1 treatment via ICV or via IN, all the measurements described below were performed.

### Behavioral Testing

Behavioral testing was performed in the Morris water maze by two investigators completely blind to animal treatment, as described in detail previously [8]. The Morris water maze (Xin Ruan XR-XM101, China), a circular black swim tank (160 cm in diameter and 50 cm in depth) with a small round escape platform (8 cm in diameter) within it, was filled with warm water (23 ± 1°C) to a depth of 27 cm and the escape platform was submerged 1 cm below the surface of the water. Before obtaining escape latency of rats in the Morris water maze, rats were given four trials (an alternation of 60 s swim and 30 s rest) per day for two consecutive days to find the hidden platform. Swimming activity of the rats was monitored by a video camera mounted overhead and automatically recorded by a video tracking system. The readout was latency to find the hidden platform, i.e., escape latency.

### Nissl Staining

Rats were perfused with 4% paraformaldehyde (pH 7.4) after anesthesia with pentobarbital (55 mg/kg, i.p.), as described in detail previously [8]. Briefly, after post-fixation in the same fixative for 2–4 h at 4°C, the brains were cut into 30 μm-thick coronal sections on a cryostat (Leica CM 1900-1-1, Germany) To ensure hippocampal sections were matched between groups, anatomical landmarks provided by the brain atlas were used. The sections were mounted on polylysine-coated slides, dried overnight, rehydrated in distilled water, and then submerged in 1% cresyl violet for about 20 min until the desired depth of staining was achieved. After being rinsed in distilled water and dehydrated in graded serried of ethanol, sections were immersed in xylene, mounted in neutral balsam, and coverslipped. Nissl-positive cells in the pyramidal layer of medial CA1 region were examined to assess neuronal loss.

### TUNEL Staining

Free-floating 40 µm coronal hippocampal sections were collected and blocked with 0.3% Triton X-100 and 3% goat serum in 0.01 M PBS (pH 7.3) for 30 min. The slices were incubated with primary antibody to NeuN produced in rabbit (Millipore, USA), diluted at 1:200 in 0.01 M PBS, overnight at room temperature. The sections were then washed in 0.01 M PBS and incubated with secondary antibody, Alexa Fluor 594 conjugated goat anti-rabbit IgG (Cell signaling Technology, USA), diluted in 0.01 M PBS (1:200), for 4 h at room temperature. Terminal deoxynucleotidyl transferase (TdT)-mediated deoxyuridine triphosphate (dUTP)-biotin nick end labeling (TUNEL) staining was performed using the In Situ Cell Death Detection Kit (Roche Applied Science, Germany), as described in detail previously [8]. Briefly, after NeuN staining, the mounted coronal sections were rinsed with PBS and treated with 1% Triton-100 in PBS for 2 min on ice. The sections were rinsed in PBS and incubated for 60 min at 37°C with 50 μl of TUNEL reaction mixture. The negative control sections were incubated for 60 min at 37°C with 50 μl of parallel solution without TdT buffer and biotinylated dUTP. After washing with PBS, the slices were analyzed with fluorescence microscopy. For each rat, a total of 15 visual fields in three hippocampal sections was counted for TUNEL-stained cells.

### Immunohistochemistry

The hippocampus was coronally cut into 40 μm-thick sections. The sections were then blocked with 0.3% Triton X-100 and 3% goat serum in 0.01 M PBS (pH 7.3) for 30 min. The slices were incubated with the primary antibodies to CD11b (AbD Serotec, UK) or to GFAP (Millipore, USA), which were diluted at 1:200 in 0.01 M PBS, overnight at room temperature. The sections were then washed in 0.01 M PBS and incubated with the secondary antibody FITC-conjugated goat anti-mouse IgG (Sigma-Aldrich, USA) diluted in 0.01 M PBS (1:200) for 4 h at room temperature. After rinsing in 0.01 M PBS, the sections were stuck to glass slides and observed under a fluorescence microscope.

### Western Blot Analysis

The hippocampus was homogenized in an SDS sample buffer that contained a mixture of proteinase inhibitors and the supernatant was collected by centrifuging at 4°C at 12,000 rpm for 15 min. Supernatants were mixed with loading buffer, which was boiled for 10 min. Proteins were separated by 10% sodium dodecyl sulfate-polyacrylamide gel electrophoresis and transferred to a polyvinylidene difluoride membrane (Pall, USA) using an electroblotting apparatus (Bio-Rad, USA). Membranes were blocked for 1 h at room temperature in Tris-buffered saline containing 0.1% Tween-20 and 5% dry milk and were then incubated with primary antibodies to APP (1:1000, Millipore, USA), PP2A (1:1000, Cell Signaling Technology, USA), tumor necrosis factor (TNF)-α (1:300, Abcam, UK), IL-1β (1:200, Santa Cruz Biotechnology, Inc., USA), inducible nitric oxide synthase (iNOS, 1:50, Abcam, UK), insulin-like growth factor (IGF)-1 (1:100, Santa Cruz Biotechnology, Inc., USA), glial-derived neurotrophic factor (GDNF, 1:100, Abcam, UK), brain-derived neurotrophic factor (BDNF, 1:100, Santa Cruz Biotechnology, Inc., USA), T-bet (1:100, Santa Cruz Biotechnology, Inc., USA), interferon (IFN)-γ (1:200, Santa Cruz Biotechnology, Inc., USA), IL-2 (1:100, Santa Cruz Biotechnology, Inc., USA), ROR-γ (1:200, Santa Cruz Biotechnology, Inc., USA), IL-17 (1:100, Santa Cruz Biotechnology, Inc., USA), IL-22 (1:100, Santa Cruz Biotechnology, Inc., USA), GATA-3 (1:100, Santa Cruz Biotechnology, Inc., USA), IL-4 (1:500, R&D Systems, Wiesbaden, Germany), Foxp3 (1:200, Santa Cruz Biotechnology, Inc., USA), or IL-10 (1:200, Santa Cruz Biotechnology, Inc., USA). The membranes were probed at 4°C overnight and incubated with IRDye 800-conjugated goat anti-rabbit IgG (1:5000, Rockland Immunochemicals, Inc., USA), IRDye 800-conjugated goat anti-mouse IgG (1:5000, Rockland Immunochemicals, Inc., USA), or IRDye 800-conjugated donkey anti-goat IgG (1:5000, LI-COR Inc, USA) for 1 h at room temperature, followed by visualization with an Odyssey laser scanning system (LI-COR Inc, USA). Blots were re-probed with monoclonal mouse anti-β-actin antibody (1:5000, Sigma-Aldrich, USA) and reacted with IRDye 800-conjugated goat anti-mouse IgG (1:5000, Rockland Immunochemicals, Inc., USA) to confirm equal protein loading. The molecular weight and relative quantity of protein bands were determined by an image analysis system (Odyssey 3.0 software).

### Real-Time PCR Analysis

Total RNA of the hippocampus was extracted with Trizol reagent (Invitrogen, USA) according to the manufacturer’s instructions, as described in detail previously [8]. Potentially contaminating residual genomic DNA was eliminated with RNAse-free DNAse (Promega, USA). After the RNA content was determined by spectrophotometric analysis at 260 nm, 2 μg of total RNA was reversely transcribed in a 20 μl reaction used for cDNA synthesis with murine myelomonocytic lymphoma virus reverse transcriptase (Promega, USA). Real-time quantitative PCR was performed on cDNA by using the Rotor-Gene 3000 Real-time Cycler with SYBR green I as the detection system. Each 20 μl of reaction mixture contained 1 μl of cDNA, 2 μl PCR buffer, 3.0 mM MgCl_2_, 0.2 mM of each dNTP, 0.2 μM of each pair of oligonucleotide primers, and 1 U Taq DNA polymerase. Reaction procedures were as follows: an initial step at 95°C for 5 min, 40 cycles of 94°C for 15 s, 62°C for 20 s, and 72°C for 20 s. Data was collected using the instrument’s software (Rotor-Gene software, version 6.0) and relative quantification was performed using the comparative threshold (CT) method after determining CT values for reference (β-actin) and target genes (TNF-α, IL-1β, iNOS, IGF-1, GDNF, BDNF, IFN-γ, IL-2, IL-17, IL-22, IL-4 or IL-10) in each sample set according to the 2^-ΔΔCt^ method [29]. Changes in mRNA expression levels were calculated after normalization to β-actin, a house-keeping gene. To verify the specificity of each amplification reaction, melting curve analyses were performed. Primer sequences for each target gene are listed in Table 1.

**Table 1:** Sequences of PCR primers.

**Gene**	**Sense primer**	**Antisense primer**
IL-2	5’-CCATGATGCTCACGTTTAAATTTT-3’	5’-CATTTTCCAGGCACTGAAGATG-3’
IFN-γ	5’-GCCCTCTCTGGCTGTTACTG-3’	5’-TACCGTCCTTTTGCCAGTTC-3’
IL-4	5’-ACCTTGCTGTCACCCTGTTCT-3’	5’-CTCTCTCAGAGGGCTGTCGTTA-3’
IL-10	5’-TGGCAACCCAAGTAACCCT-3’	5’-CACCCACTTCCCAGTCAGC-3’
IL-17	5’-TGGACTCTGAGCCGCAATG-3’	5’-GGCGGACAATAGAGGAAACG-3’
IL-22	5’-AGCGGTGATGACCAGAACA-3’	5’-CTCAGGGACATAAACAGCAGA-3’
iNOS	5’-CAGCTGGGCTGTACAAACCTT-3’	5’-CATTGGAAGTGAAGCGTTTCG-3’
IL-1β	5’-CTTCCTTGTGCAAGTGTCTG-3’	5’-CAGGTCATTCTCATCACTGTC-3’
TNF-α	5’-CCACCACGCTCTTCTGTCTAC-3’	5’-ATCTGAGTGTGGGGTCTGG-3’
IGF-1	5’-TTTTACTTCAACAAGCCCACA-3’	5’-CATCCACAATGCCCGTCT-3’
GDNF	5’- ATTCAAGCCACCATCAAAAG-3’	5’-TCAGTTCCTCCTTGGTTTCG-3’
BDNF	5’-ATCCCATGGGTTACACGAAGGAAG-3’	5’-AGTAAGGGCCCGAACATACGATTG-3’
β-actin	5’-CGTTGACATCCGTAAAGACC-3’	5’-TAGAGCCACCAATCCACAC-3’

### Enzyme-Linked Immunosorbent Assay (ELISA)

As described in detail previously [8], blood was taken from the right ventricle, and serum was collected by centrifugation at 3,000 rpm for 20 min. Cerebrospinal fluid (CSF) was withdrawn by foramen magnum puncture. Serum and CSF were stored at -80°C until use. Concentrations of the target cytokines, IFN-γ, IL-1β, IL-17 and IL-10, in serum and/or CSF were measured by ELISA kits (eBioscience, USA) according to the manufacturers’ guidelines.

### Statistical Analysis

Data were expressed as mean values ± standard deviation of each group. Statistical analysis was carried out with the Statistics Package for Social Science (SPSS, 12.0). The data were analyzed with one-way analysis of variance (ANOVA) for comparison of the differences between the various groups. Following ANOVA, the Student-Newman-Keul’s test was used for pairwise comparisons of data. Differences were considered statistically significant at *p* < 0.05.

## Results

### ICV and IN Administered TGF-β1 Both Reduce Aβ_1–42_-Induced Cognitive Impairment and Neurotoxicity

**Figure 1:**
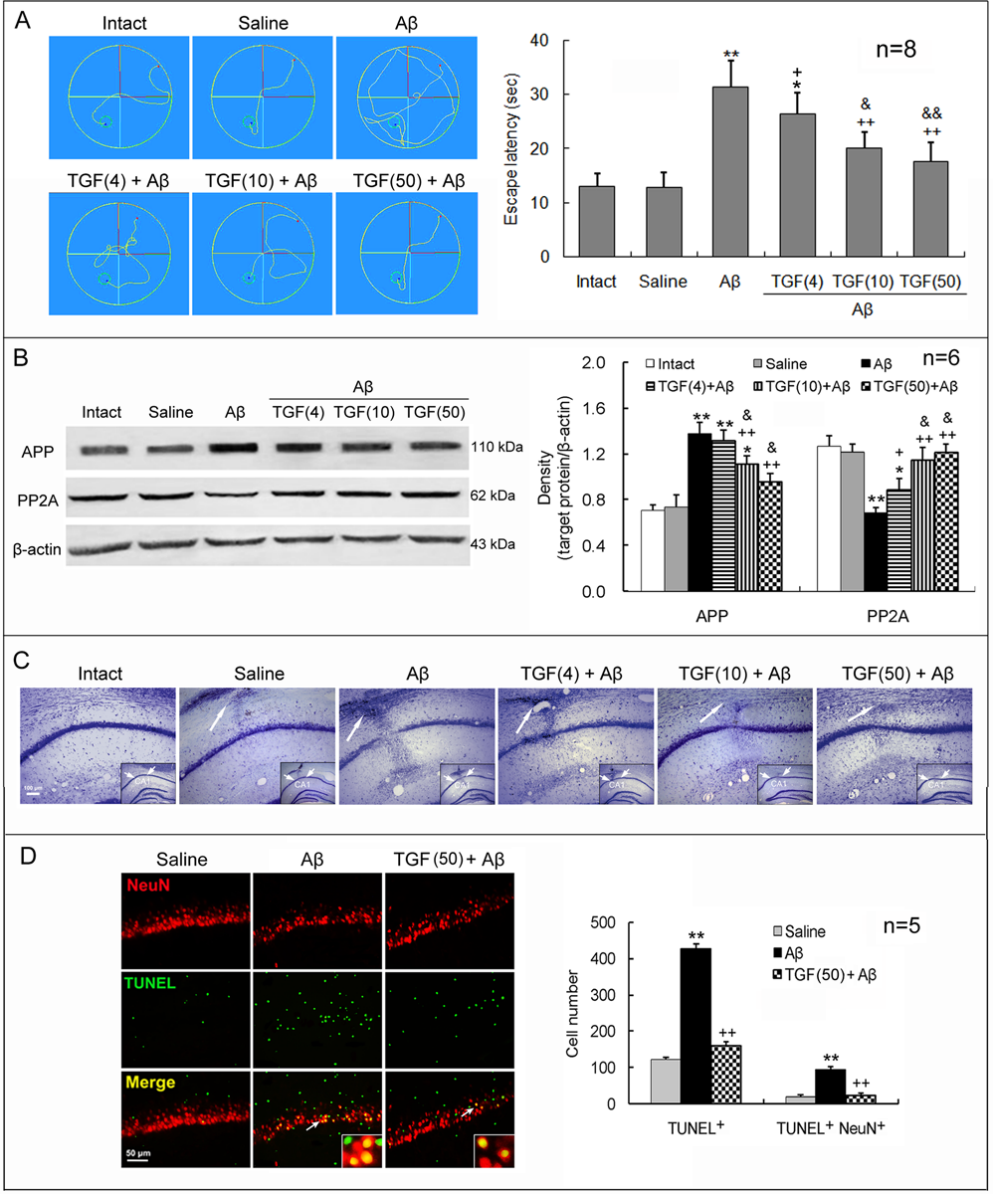
ICV TGF-β1 pretreatment prevents Aβ_1–42_-induced cognitive impairment and neuronal loss and apoptosis. TGF-β1 (4, 10 or 50 ng in 5 μl) was given ICV one hour prior to bilateral hippocampal Aβ_1–42_ injection in rats. On day 7 following TGF-β1 administration, behavioral and neuronal changes were measured. **(A)** Escape latency in Morris water maze. Left panel exhibits swimming tracks of rats in Morris water maze. The escape latency was recorded from the time the rats entered the water (the red points) until their arrival on the platform (the green small rounds). Right panel is a statistical histogram for these experiments. **(B)** Expression levels of APP and PP2A in the hippocampus. Left panel shows representative electrophoretic bands, and right panel indicates graphical results of repeated experiments. **(C)** Nissl stain of hippocampal CA1 region of rats. The big arrows point to locations where injection needles were placed and reactive gliosis is seen. Note that Aβ_1–42_ induced an obvious neuron loss in the CA1 region and that TGF-β1 reduced this loss, reflected by the density of cells with Nissl bodies. The insets within the images are general views of the hippocampus, in which the CA1 region is denoted. **(D)** Immunofluorescent histochemistry for NeuN and TUNEL in the hippocampus. Left panel is a representative image. The arrows point to the NeuN/TUNEL double-stained cells, which are magnified in the insets. Right panel is a statistical histogram of the repeated experiments. For each rat, a total of 15 visual fields in three hippocampal sections was counted for the TUNEL-positive cells. Aβ = Aβ_1–42_; TGF(4) = 4 ng of TGF-β1; TGF(10) = 10 ng of TGF-β1; TGF(50) = 50 ng of TGF-β1. *p<0.05, **p<0.01, versus intact or saline-treated rats; +*p*<0.05, ++*p*<0.01, versus alone Aβ_1–42_-injected rats; &*p*<0.05, &&*p*<0.01, versus 4 or 10 ng of TGF-β1 administered rats.

TGF-β1 (4, 10 or 50 ng in 5 μl) was injected into the left lateral cerebral ventricle of rats one hour prior to bilateral hippocampal Aβ_1–42_ injection. On day 7 following these treatments, AD-associated changes in these rats were measured. Aβ_1–42_ injection significantly increased escape latency in Morris water maze, upregulated APP expression, downregulated PP2A expression, and led to a loss of Nissl bodies and an elevation of NeuN/TUNEL double-positive cell number in the CA1 region, with respect to intact or saline-injected rats (Fig. 1). In addition to the cognitive and pathological impairments, the Aβ_1–42_ injection also induced clinical manifestations, including reduced attention, weak activeness, and depressed exploration behavior. Significantly, compared with Aβ_1–42_ injection alone, TGF-β1 ICV pretreatment combined with the Aβ_1–42_ injection decreased escape latency, downregulated APP expression, upregulated PP2A expression, and reduced apoptotic neuronal number (Fig. 1). These TGF-β1 effects were concentration-dependent.

**Figure 2:**
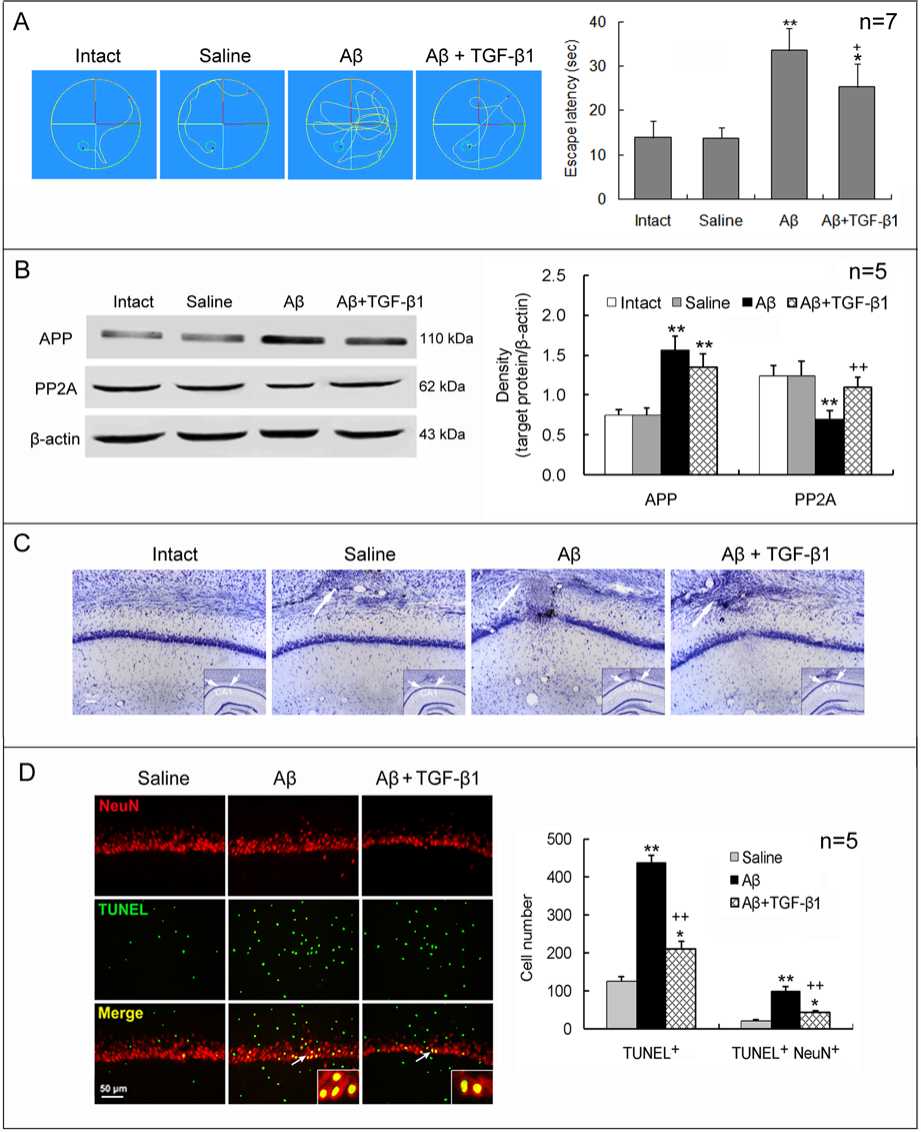
IN TGF-β1 post-treatment reduces Aβ_1–42_-induced cognitive impairment and neuronal loss and apoptosis. TGF-β1 (1.5 μg/30 μl) was given IN seven days after Aβ_1–42_ injection. On day 7 following TGF-β1 administration, escape latency in Morris water maze (A), expression of APP and PP2A (B), and neuronal loss (C) and apoptosis (D) in the hippocampus were measured. The design of the experiments and the meaning of the figure are similar to those of Fig. 1, except that TGF-β1 was given IN after Aβ_1–42_ injection at one concentration. *p<0.05, **p<0.01, versus intact or saline-treated rats; +*p*<0.05, ++*p*<0.01, versus alone Aβ_1–42_-injected rats.

To confirm the neuroprotective effect of TGF-β1 against Aβ_1–42_ toxicity, we used a second approach to administer TGF-β1, in which TGF-β1 (1.5 μg/30 μl) was given via both nares on the 7th day after Aβ_1–42_ injection. IN administration of TGF-β1 still remarkably reduced the increased escape latency, the downregulated PP2A and the increased neuronal loss and apoptosis induced by Aβ_1–42_, but did not significantly reduce Aβ_1–42_-induced upregulation of APP expression (Fig. 2).

### ICV and IN Administered TGF-β1 Both Inhibit Aβ_1–42_-Induced Activation of Glial Cells

**Figure 3:**
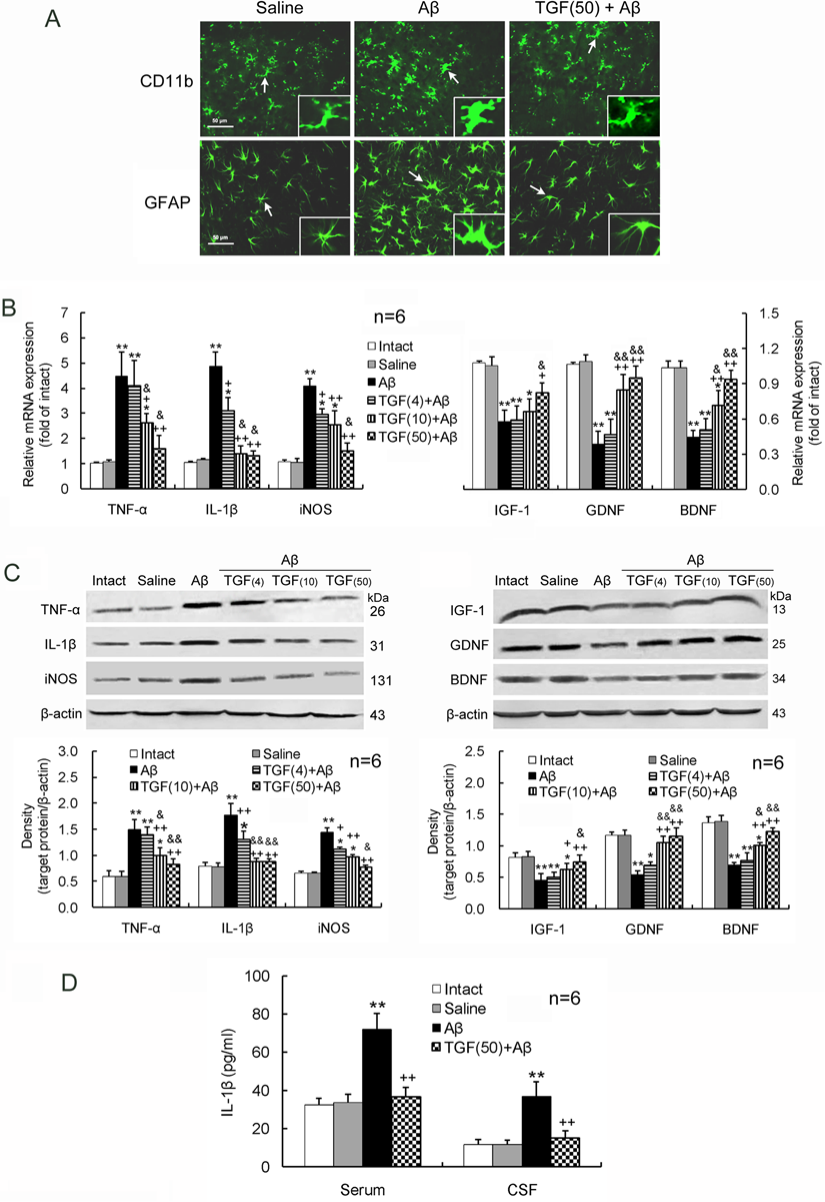
ICV TGF-β1 pretreatment prevents Aβ_1–42_-induced glial activation. TGF-β1 (4, 10 or 50 ng in 5 μl) was given ICV one hour prior to Aβ_1–42_ injection. On day 7 following TGF-β1 administration, glial cells including microglia and astrocytes in the hippocampus were evaluated for their activation and function. **(A)** Immunofluorescent histochemistry of hippocampal sections. Note that the microglia and astrocytes, tagged by CD11b and GFAP, respectively, are obviously increased in soma size with retraction of processes by the Aβ_1–42_ treatment. The characteristics meet morphological criteria of activation of microglia and astrocytes. TGF-β1 pretreatment before Aβ_1–42_ injection reduces soma size of microglia and astrocytes compared with Aβ_1–42_ injection alone. The insets are amplifications of the cells indicated by arrows. (B) mRNA expression levels of the proinflammatory mediators, TNF-α, IL-1β and iNOS, and the neurotrophic factors, IGF-1, GDNF and BDNF, in the hippocampus. (C) Protein expression levels of the proinflammatory mediators and the neurotrophic factors. (D) IL-1β concentrations in the serum and CSF measured by ELISA. *p<0.05, **p<0.01, versus intact or saline-treated rats; +*p*<0.05, ++*p*<0.01, versus alone Aβ_1–42_-injected rats; &*p*<0.05, &&*p*<0.01, versus 4 or 10 ng of TGF-β1 administered rats. Aβ = Aβ_1–42_; TGF(4) = 4 ng of TGF-β1; TGF(10) = 10 ng of TGF-β1; TGF(50) = 50 ng of TGF-β1.

Aβ_1–42_ induced an upregulation of the inflammatory mediators, TNF-α, IL-1β and iNOS, and a downregulation of the neurotrophic factors, IGF-1, GDNF and BDNF, at gene and protein expression levels in the hippocampus (Fig. 3A-B). Simultaneously, Aβ_1–42_ caused an elevation of the inflammatory mediator IL-1β in the serum and CSF (Fig. 3C). TGF-β1 (4, 10 or 50 ng in 5 μl) administered ICV prior to Aβ_1–42_ injection inhibited all the Aβ_1–42_-induced changes in a concentration-dependent manner (Fig. 3).

**Figure 4:**
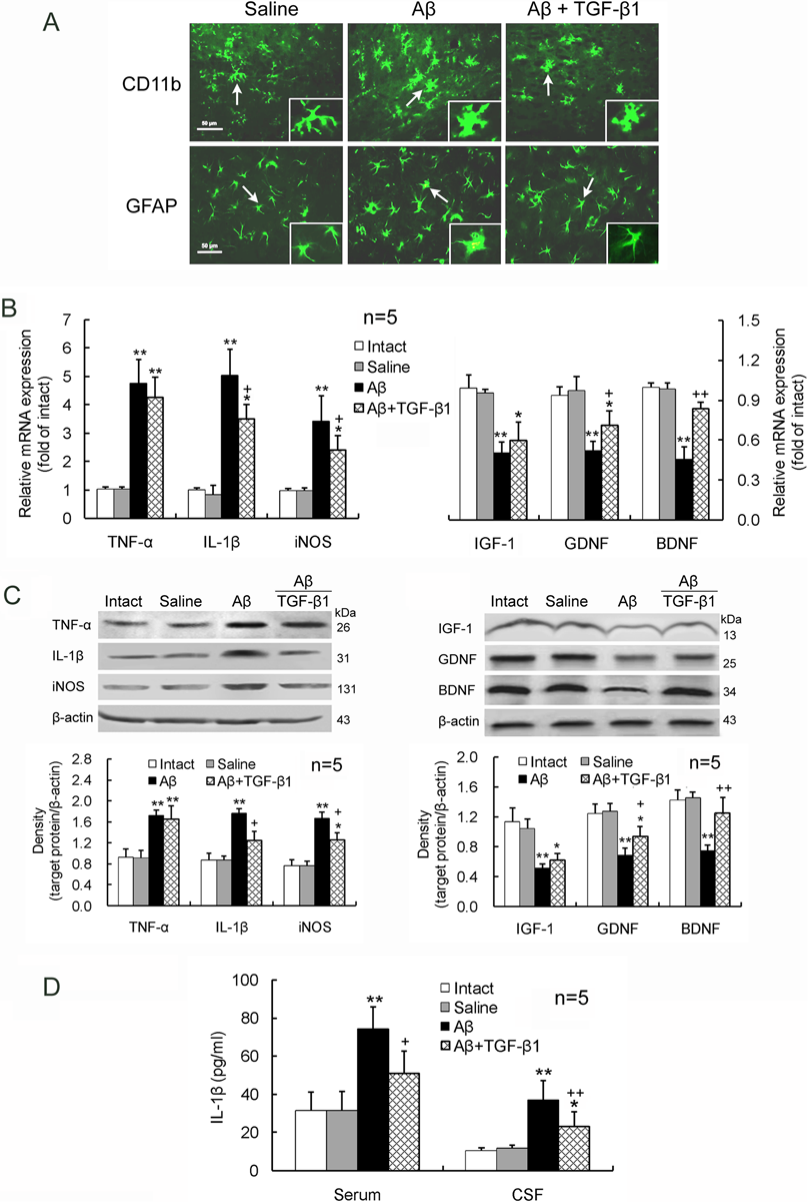
IN TGF-β1 post-treatment alleviates Aβ_1–42_-induced glial activation. TGF-β1 (1.5 μg/30 μl) was given IN seven days after Aβ_1–42_ injection. On day 7 following TGF-β1 administration, glial activation morphology (A), gene (B) and protein (C) expression levels of the proinflammatory mediators and neurotrophic factors in the hippocampus, as well as IL-1β concentrations in the serum and CSF (D) were measured. The design of the experiments and the meaning of the figure are similar to those of Fig. 3, except that TGF-β1 was given IN after Aβ_1–42_ invasion at one concentration. *p<0.05, **p<0.01, versus intact or saline-treated rats; +*p*<0.05, ++*p*<0.01, versus alone Aβ_1–42_-injected rats.

In addition, IN TGF-β1 treatment (1.5 μg/30 μl) after Aβ_1–42_ injection reduced the increased IL-1β and iNOS expression and the decreased GDNF and BDNF expression in the hippocampus (Fig. 4A-B). But TGF-β1 treatment did not significantly alter Aβ_1–42_-induced upregulation of TNF-α or downregulation of IGF-1 in the hippocampus (Fig. 4A-B). Elevated IL-1β concentrations in the serum and CSF by Aβ_1–42_ were reduced by the IN TGF-β1 administration (Fig. 4C).

### ICV and IN Administered TGF-β1 Both Alleviate Aβ_1–42_-Induced Proinflammatory Enhancement and Antiinflammatory Attenuation of T Lymphocytes

**Figure 5:**
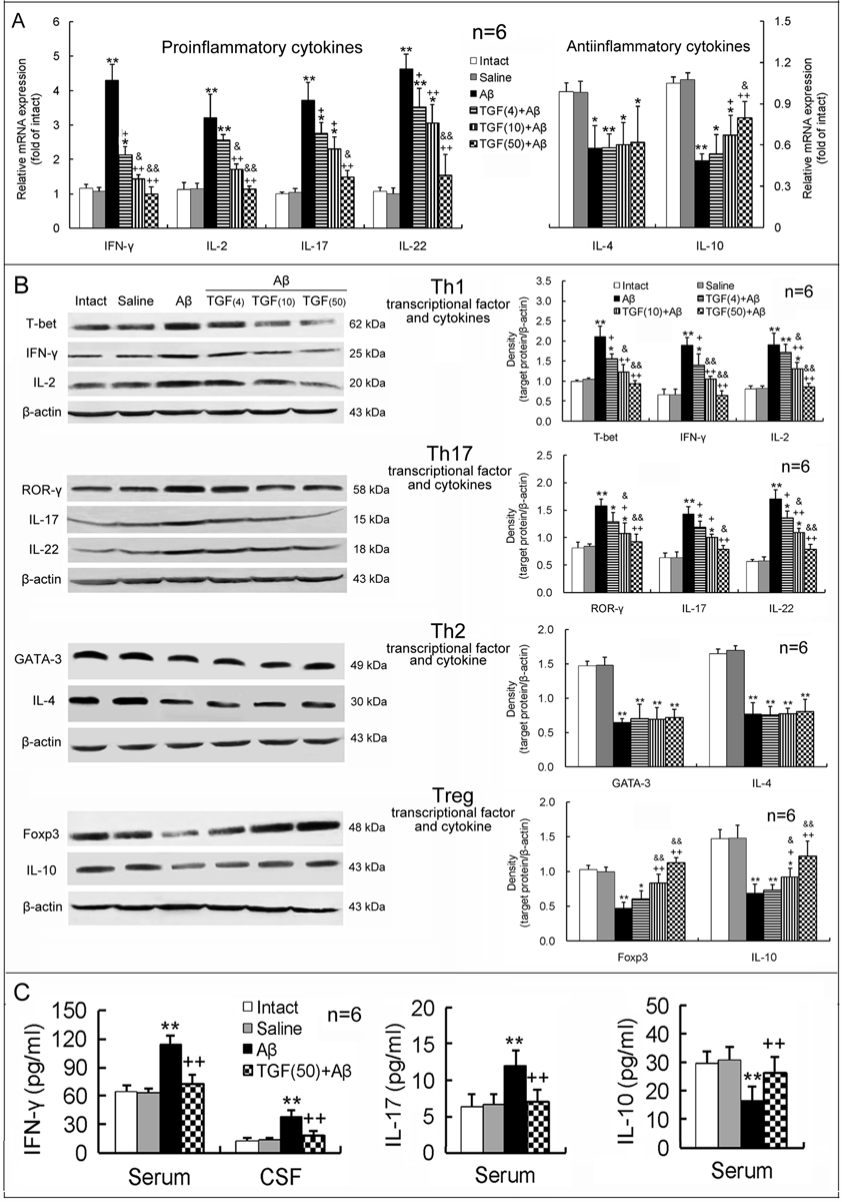
ICV TGF-β1 pretreatment prevents Aβ_1–42_-induced imbalance in proinflammatory/antiinflammatory responses of T lymphocytes. TGF-β1 (4, 10 or 50 ng in 5 μl) was given ICV one hour prior to Aβ_1–42_ injection. On day 7 following TGF-β1 administration, differentiation and function of Th1, Th17, Th2 and Treg cells were assessed by measuring levels of specific transcriptional factors and cytokines in the hippocampus, serum and CSF. **(A)** Gene expression of T lymphocyte-related proinflammatory and antiinflammatory cytokines in the hippocampus. **(B)** Protein expression of specific transcriptional factors (T-bet, ROR-γ, GATA-3 and Foxp3) and cytokines of T lymphocyte subsets in the hippocampus. **(C)** Concentrations of Th1- and Th17-related proinflammatory cytokines (IFN-γ and IL-17) and the Treg-related antiinflammatory cytokine (IL-10) in serum and/or CSF. *p<0.05, **p<0.01, versus intact or saline-treated rats; +*p*<0.05, ++*p*<0.01, versus alone Aβ_1–42_-injected rats; &*p*<0.05, &&*p*<0.01, versus 4 or 10 ng of TGF-β1 administered rats. Aβ = Aβ_1–42_; TGF(4) = 4 ng of TGF-β1; TGF(10) = 10 ng of TGF-β1; TGF(50) = 50 ng of TGF-β1.

As shown in Fig. 5A and B, Aβ_1–42_ strikingly upregulated mRNA and protein expression levels of the proinflammatory cytokines, IFN-γ, IL-2, IL-17 and IL-22, but downregulated expression levels of the antiinflammatory cytokines, IL-4 and IL-10, in the hippocampus. Simultaneously, expression levels of T-bet and ROR-γ, the specific transcription factors associated with proinflammatory Th1 and Th17 cells, respectively, were upregulated, whereas expression levels of GATA-3 and Foxp3, the specific transcription factors associated with antiinflammatory Th2 and Treg cells, respectively, were downregulated in the hippocampus of AD model rats (Fig. 5B). Moreover, in the serum or CSF of AD rats, concentrations of the proinflammatory cytokines IFN-γ and IL-17 were elevated, while the concentration of the antiinflammatory cytokine IL-10 was decreased, with respect to those of intact or saline-injected animals (Fig. 5C). Notablely, pretreatment with TGF-β1 (4, 10 or 50 ng in 5 μl) via ICV before Aβ_1–42_ injection inhibited Th1- and Th17-proinflammatory responses and enhanced Treg-antiinflammatory response in the hippocampus, serum and CSF, in comparison to the treatment with Aβ_1–42_ alone (Fig. 5). These TGF-β1 effects were concentration-dependent. However, TGF-β1 pretreatment did not prevent the Aβ_1–42_ injection-induced decrease observed in GATA-3 and IL-4, the transcription factor and cytokine of antiinflammatory Th2 cells, respectively, in the hippocampus (Fig. 5A-B).

**Figure 6:**
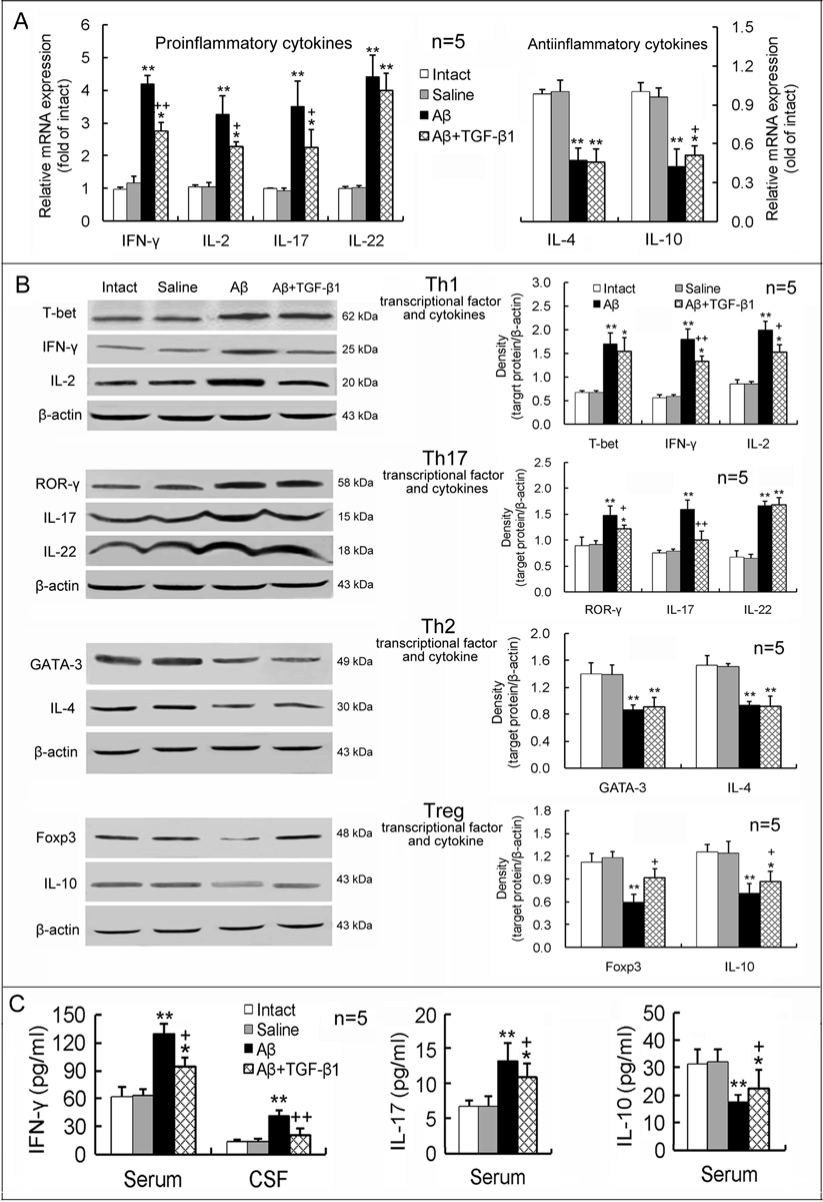
IN TGF-β1 post-treatment ameliorates Aβ_1–42_-induced imbalance in proinflammatory/antiinflammatory responses of T lymphocytes. TGF-β1 (1.5 μg/30 μl) was given IN seven days after Aβ_1–42_ injection. On the 7th day following TGF-β1 administration, differentiation and function of Th1, Th17, Th2 and Treg cells were measured. The design of the experiments and meaning of the figure are similar to those of Fig. 5, except that TGF-β1 was given IN after Aβ_1–42_ injection at one concentration. *p<0.05, **p<0.01, versus intact or saline-treated rats; +*p*<0.05, ++*p*<0.01, versus alone Aβ_1–42_-injected rats.

Furthermore, TGF-β1 treatment via IN after Aβ_1–42_ injection significantly reduced expression of IFN-γ, IL-2, IL-17 and ROR-γ and also elevated expression of IL-10 and Foxp3 in the hippocampus, with respect to Aβ_1–42_ injection alone (Fig. 6A-B). But upregulated IL-22 and T-bet and downregulated IL-4 and GATA-3 expression levels induced by Aβ_1–42_ were not significantly affected by TGF-β1 post-treatment via IN (Fig. 6A-B). Simultaneously, elevated levels of the proinflammatory cytokines, IFN-γ and IL-17, and diminished level of the antiinflammatory cytokine, IL-10, in the serum or CSF induced by Aβ_1–42_ were ameliorated by IN TGF-β1 post-treatment (Fig. 6C).

## Discussion

It has been shown that intrahippocampal Aβ_1–42_ injection has many characteristics similar to those found in AD brain [30]. Therefore, Aβ_1–42_ injection is a useful animal model of AD [6, 30]. We have recently reported that Aβ_1–42_ injection in bilateral hippocampus induces a cognitive impairment, BBB disruption, and neuronal apoptosis on both the 7th and 14th days post-Aβ_1–42_ injection [8]. In the present study, we confirm that the intrahippocampal Aβ_1–42_ injection induces an AD-associated neurodegeneration. Importantly, TGF-β1 pretreatment via ICV prevented Aβ_1–42_-induced behavioral and pathological impairments in a concentration-dependent manner. These results suggest that TGF-β1 can be neuroprotective against AD initiation. Since the preventive effect of TGF-β1 via ICV administration on AD initiation could not be implemented in actual patients, treatment with IN TGF-β1 after Aβ_1–42_ injection was performed. Although increased APP expression in the hippocampus of AD model rats was not significantly reduced, cognitive impairment, PP2A downregulation and neuronal loss and apoptosis were alleviated by TGF-β1 post-treatment via IN. These data provide strong evidence for the potential therapeutic application of TGF-β1 in AD progression.

The administration of TGF-β1 via ICV has been reported by Henrich-Noack et al. [31]. They show that ICV administration of 4 ng TGF-β1 one hour before ischemia reduces percentage of damaged CA1 pyramidal cells and this effect is the same as that obtained by injection of 4 ng TGF-β1 directly into hippocampal tissue. These data suggest TGF-β1 ICV administration can deliver it into the brain to exert the same neuroprotection as brain parenchymal injection. In addition, Ma et al. [32] have shown that IN administration of TGF-β1 significantly raises its concentrations in several brain regions, such as the olfactory bulb (with the highest concentration), olfactory tubercle, striatum, cortex, thalamus, hippocampus and medulla, and in the trigeminal nerve. Therefore, they suggest that TGF-β1 can be transported into the central nervous system via the olfactory and trigeminal pathways. TGF-β1 has been reported to be immunosuppressive and neuroprotective [33], but its role in AD is less clear. Here, we show that TGF-β1 has a preventive or therapeutic effect on AD-associated impairments when given exogenously in vivo. Although the TGF-β1 IN therapeutic effect may not be so large as the TGF-β1 ICV preventive effect, the effective and safe IN administrative approach provides a practical paradigm for treating AD patients with TGF-β1.

Effects of TGF-β1 depend on its signaling. An impairment of TGF-β1 signaling pathway is specific to AD brain and, particularly, to early phase of the disease [34, 35]. The deficiency of TGF-β1 signaling is associated with Aβ pathology and neurofibrillary tangle formation in AD animal models and reduced TGF-β1 signaling seems to contribute both to microglial activation and to ectopic cell-cycle re-activation in neurons, two events that contribute to neurodegeneration in AD brain [36]. Therefore, rescuing TGF-β1 signaling is critical for TGF-β1 neuroprotective role. It has been reported that nasally administered TGF-β1 upregulates gene expressions of its two receptors (TGF-β receptor types I and II) in the brain, but does not affect mRNA level of TGF-β1 itself, suggesting that TGF-β1 may exert neuroprotective property by activation of its signaling in the brain [32]. Accordingly, our present results imply that the exogenously given TGF-β1 slows down AD-associated neurodegeneration by rescuing TGF-β1 signaling.

In addition to the conventional manifestations of AD, neuroinflammation also plays a pathogenic role in AD [37, 38]. A major characteristic of AD neuroinflammation is microglial and astrocytic activation by Aβ deposits, with subsequent production of proinflammatory cytokines and chemokines that recruit more neurotoxic glial cells and lead to further neuroinflammation and neurotoxicity [2, 3, 39–41]. In the present study, we observed increased expression of the proinflammatory mediators, TNF-α, IL-1β and iNOS, and decreased expression of the neurotrophic factors, IGF-1, GDNF and BDNF, in the hippocampus of AD model rats. Since the proinflammatory mediators and neurotrophic factors are produced primarily by microglia and astrocytes in the brain, these findings confirm an activation of glial cells in AD brain. Importantly, TGF-β1 administration by both ICV and IN routes inhibited the activation of glial cells induced by Aβ_1–42_. Although these effects of TGF-β1 were greater in the ICV pretreatment protocol than in the IN post-treatment approach, the effectiveness of the IN administration in inhibiting glial activation provides further support for the idea of treating AD patients with TGF-β1.

Glia-mediated neuroinflammation has long-been recognized in AD pathogenesis. In addition, changes in peripheral T cells in AD patients or in AD animal models have been also reported. For example, a significant increase in CD4^+^, CD25^+^ and CD28^+^ cells in blood mononuclear cells is observed in AD patients [42]. A increased reactivity of peripheral CD4^+^ and CD8^+^ T cells to mitogen is found in AD patients [43]. The triple transgenic (3xTg-AD) mice have increased percentages of dendritic cells and macrophages, spleen and blood derived CD8^+^Ly6C^+^ memory T cells and CCR6^+^ B cells [44]. These findings suggest that peripheral T cell-mediated immunity is involved in AD pathogenesis. The first evidence that T cells were present in the brain of AD patients was presented 25 years ago and similar findings have been sporadically reported since [45]. These cells were found to be in close apposition with plaques and activated glia [46]. In the brains of APP/PS1 mice, there also is significant infiltration of T cells, particularly IFN-γ-positive and IL-17-positive T cells [47]. In the current study, we found in peripheral blood and in central CSF and hippocampus of AD model rats that Th1- and Th17-proinflammatory responses were enhanced, while Th2- and Treg-antiinflammatory responses were attenuated. The imbalance of proinflammation/antiinflammation represents an evident inflammatory reaction in our AD model. TGF-β1 administered in two different ways reduced the Th1- and Th17-proinflammatory responses and elevated the Treg-antiinflammatory response in AD model rats. However, neither TGF-β1 treatment increased Aβ_1–42_-induced downregulation of GATA-3 or IL-4 expression, suggesting that TGF-β1 does not improve Aβ_1–42_-suppressed Th2 response. This phenomenon may be related to TGF-β1 itself inhibition of Th2 development [48]. Nevertheless, the notable suppression of proinflammatory responses by either TGF-β1 treatment indicates a reduction of the inflammatory reaction and a recovery towards balance of proinflammation/antiinflammation.

The mechanism underlying peripheral T cell infiltration into AD brain parenchyma may be BBB dysfunction. We recently provide direct evidence showing that Th17 cells infiltrate into brain parenchyma through disrupted BBB in AD model rats [8]. Intracerebral Aβ interaction with its receptor at BBB upregulates endothelial CCR5 expression and causes circulating T cell infiltration into the brain induced by Aβ injection in rat hippocampus [49]. Microglia-derived TNF-α plays a crucial role in the peripheral T cell infiltration. This TNF-α upregulates MHC class I molecule expression on brain endothelial cells and induces CXCR2 overexpression in peripheral T cells in AD, which represents a mechanism of T cell migration into the brain [50, 51]. These mechanisms may be also appropriate for explanation of the peripheral T cell activation by the intrahippocampal injection of Aβ_1–42_ in this study. In addition, although there are no conventional lymphatics in the brain, both interstitial fluid and CSF have well defined lymphatic drainage pathways, which drain fluid and solutes from the brain to cervical lymph nodes [52]. Through the pathways, intracerebral TNF-α may filtrate out of the brain into the periphery to activate peripheral T cells.

Neuroinflammation is an active process detectable in the earliest stages of AD that might be a critical contributor to neurofibrillary tangle formation, one of crucial pathological hallmarks of AD [53]. As an immunosuppressive cytokine, the most likely explanation for TGF-β1’s ability to protect neurons is by inhibiting neuroinflammation mediated by glial cells and T lymphocytes. This hypothesis may explain the better outcome of TGF-β1 prevention than TGF-β1 treatment, because the TGF-β1 preventive strategy targets initiation of neuroinflammation. Therefore, inhibition of neuroinflammation is an important target for TGF-β1 prevention and treatment of AD neurodegeneration. However, the relationship between neuroinflammation and neurodegeneration in AD pathogenesis is complicated and the sequence of neuroinflammation and neurodegeneration that leads to AD is not full clarified. Thus, mechanisms underlying TGF-β1 prevention and treatment of AD still remain to be defined.

In summary, Aβ_1–42_ induces marked neuroinflammation and neurodegeneration in the rat brain. The neuroinflammation includes activation of glial cells and imbalance in proinflammatory/antiinflammatory responses of T lymphocytes. The neurodegeneration manifests as cognitive impairment, APP upregulation, PP2A downregulation, and neuronal loss and apoptosis in the hippocampus. TGF-β1 given by two methods, ICV prior to Aβ_1–42_ injection and IN after Aβ_1–42_ injection, inhibits the neuroinflammatory response and alleviates neurodegeneration. These findings suggest that TGF-β1 has both preventive and therapeutic effects on the occurrence and progression of AD-associated pathology. In particular, the effectiveness of TGF-β1 via IN administration provides a promising therapeutic strategy for patients with AD.

## References

[1] Heneka MT, O’Banion MK, Terwel D, Kummer MP (2010) Neuroinflammatory processes in Alzheimer’s disease. J Neural Transm 117: 919–947. 10.1007/s00702-010-0438-z 20632195

[2] Rubio-Perez JM, Morillas-Ruiz JM (2012) A review: inflammatory process in Alzheimer’s disease, role of cytokines. Scientific World Journal 2012: 756357 10.1100/2012/756357 22566778 PMC3330269

[3] Rojo LE, Fernández JA, Maccioni AA, Jimenez JM, Maccioni RB (2008) Neuroinflammation: implications for the pathogenesis and molecular diagnosis of Alzheimer’s disease. Arch Med Res 39: 1–16. 10.1016/j.arcmed.2007.10.001 18067990

[4] Schwab C, McGeer PL (2008) Inflammatory aspects of Alzheimer disease and other neurodegenerative disorders. J Alzheimers Dis 13: 359–369. 18487845 10.3233/jad-2008-13402

[5] Ryu JK, McLarnon JG (2008) Thalidomide inhibition of perturbed vasculature and glial-derived tumor necrosis factor-alpha in an animal model of inflamed Alzheimer’s disease brain. Neurobiol Dis 29: 254–266. 10.1016/j.nbd.2007.08.019 17964176

[6] Ryu JK, McLarnon JG (2009) A leaky blood-brain barrier, fibrinogen infiltration and microglial reactivity in inflamed Alzheimer’s disease brain. J Cell Mol Med 13: 2911–2925. 10.1111/j.1582-4934.2008.00434.x 18657226 PMC4498946

[7] Saresella M, Calabrese E, Marventano I, Piancone F, Gatti A, et al (2011) Increased activity of Th-17 and Th-9 lymphocytes and a skewing of the post-thymic differentiation pathway are seen in Alzheimer’s disease. Brain Behav Immun 25: 539–547. 10.1016/j.bbi.2010.12.004 21167930

[8] Zhang J, Ke KF, Liu Z, Qiu YH, Peng YP (2013) Th17 cell-mediated neuroinflammation is involved in neurodegeneration of abeta 1–42-induced Alzheimer’s disease model rats. PLoS One 8: e75786 10.1371/journal.pone.0075786 24124514 PMC3790825

[9] Ten Dijke P, Hill CS (2004) New insights into TGF-beta-Smad signalling. Trends Biochem Sci 29: 265–273. 10.1016/j.tibs.2004.03.008 15130563

[10] Li MO, Wan YY, Sanjabi S, Robertson AK, Flavell RA (2006) Transforming growth factor-beta regulation of immune responses. Annu Rev Immunol 24: 99–146. 10.1146/annurev.immunol.24.021605.090737 16551245

[11] Dhandapani KM, Hadman M, De Sevilla L, Wade MF, Mahesh VB, et al (2003) Astrocyte protection of neurons: role of transforming growth factor-beta signaling via a c-Jun-AP-1 protective pathway. J Biol Chem 278: 43329–43339. 10.1074/jbc.M305835200 12888549

[12] Vivien D, Ali C (2006) Transforming growth factor-beta signalling in brain disorders. Cytokine Growth Factor Rev 17: 121–128. 10.1016/j.cytogfr.2005.09.011 16271500

[13] Wyss-Coray T (2006) Tgf-Beta pathway as a potential target in neurodegeneration and Alzheimer’s. Curr Alzheimer Res 3: 191–195. 10.2174/156720506777632916 16842094

[14] Caraci F, Battaglia G, Busceti C, Biagioni F, Mastroiacovo F, et al (2008) TGF-beta 1 protects against Abeta-neurotoxicity via the phosphatidylinositol-3-kinase pathway. Neurobiol Dis 30: 234–242. 10.1016/j.nbd.2008.01.007 18356065

[15] Caraci F, Battaglia G, Bruno V, Bosco P, Carbonaro V, et al (2011) TGF-beta1 pathway as a new target for neuroprotection in Alzheimer’s disease. CNS Neurosci Ther 17: 237–249. 10.1111/j.1755-5949.2009.00115.x 19925479 PMC6493850

[16] Juraskova B, Andrys C, Holmerova I, Solichova D, Hrnciarikova D, et al (2010) Transforming growth factor beta and soluble endoglin in the healthy senior and in Alzheimer’s disease patients. J Nutr Health Aging 14: 758–761. 10.1007/s12603-010-0325-1 21085906 PMC12880356

[17] Luppi C, Fioravanti M, Bertolini B, Inguscio M, Grugnetti A, et al (2009) Growth factors decrease in subjects with mild to moderate Alzheimer’s disease (AD): potential correction with dehydroepiandrosterone-sulphate (DHEAS). Arch Gerontol Geriatr 49: 173–184. 10.1016/j.archger.2009.09.027 19836631

[18] Wyss-Coray T, Lin C, Yan F, Yu GQ, Rohde M, et al (2001) TGF-beta1 promotes microglial amyloid-beta clearance and reduces plaque burden in transgenic mice. Nat Med 7: 612–618. 10.1038/87945 11329064

[19] Thorne RG, Frey WH (2001) Delivery of neurotrophic factors to the central nervous system: pharmacokinetic considerations. Clin Pharmacokinet 40: 907–946. 10.2165/00003088-200140120-00003 11735609

[20] Thorne RG, Pronk GJ, Padmanabhan V, Frey WH (2004) Delivery of insulin-like growth factor-I to the rat brain and spinal cord along olfactory and trigeminal pathways following intranasal administration. Neuroscience 127: 481–496. 10.1016/j.neuroscience.2004.05.029 15262337

[21] Ma M, Ma Y, Yi X, Guo R, Zhu W, et al (2008) Intranasal delivery of transforming growth factor-beta1 in mice after stroke reduces infarct volume and increases neurogenesis in the subventricular zone. BMC Neurosci 9: 117 10.1186/1471-2202-9-117 19077183 PMC2637876

[22] Morales I, Farías G, Maccioni RB (2010) Neuroimmunomodulation in the pathogenesis of Alzheimer’s disease. Neuroimmunomodulation 17: 202–204. 10.1159/000258724 20134203

[23] McLarnon JG, Ryu JK (2008) Relevance of abeta1–42 intrahippocampal injection as an animal model of inflamed Alzheimer’s disease brain. Curr Alzheimer Res 5: 475–480. 10.2174/156720508785908874 18855589

[24] Pike CJ, Burdick D, Walencewicz AJ, Glabe CG, Cotman CW (1993) Neurodegeneration induced by beta-amyloid peptides in vitro: the role of peptide assembly state. J Neurosci 13: 1676–1687. 8463843 10.1523/JNEUROSCI.13-04-01676.1993 PMC6576726

[25] Giuffrida ML, Grasso G, Ruvo M, Pedone C, Saporito A, et al (2007) Abeta(25–35) and its C-and/or N-blocked derivatives: copper driven structural features and neurotoxicity. J Neurosci Res 85: 623–633. 10.1002/jnr.21135 17131391

[26] Paxinos G, Watson C (2005) The Rat Brain in Stereotaxic Coordinates. Amsterdam: Elsevier Academic Press.

[27] Liu XF, Fawcett JR, Thorne RG, DeFor TA, Frey WH (2001) Intranasal administration of insulin-like growth factor-I bypasses the blood-brain barrier and protects against focal cerebral ischemic damage. J Neurol Sci 187: 91–97. 10.1016/S0022-510X(01)00532-9 11440750

[28] Liu XF, Fawcett JR, Thorne RG, Frey WH (2001) Non-invasive intranasal insulin-like growth factor-I reduces infarct volume and improves neurologic function in rats following middle cerebral artery occlusion. Neurosci Lett 308: 91–94. 10.1016/S0304-3940(01)01982-6 11457567

[29] Livak KJ, Schmittgen TD (2001) Analysis of relative gene expression data using real-time quantitative PCR and the 2(-Delta Delta C(T)) Method. Methods 25: 402–408. 10.1006/meth.2001.1262 11846609

[30] Jantaratnotai N, Ryu JK, Schwab C, McGeer PL, McLarnon JG (2011) Comparison of Vascular Perturbations in an Abeta-Injected Animal Model and in AD Brain. Int J Alzheimers Dis 2011: 918280 10.4061/2011/918280 21969915 PMC3182764

[31] Henrich-Noack P, Prehn JH, Krieglstein J (1996) TGF-beta 1 protects hippocampal neurons against degeneration caused by transient global ischemia. Dose-response relationship and potential neuroprotective mechanisms. Stroke 27: 1609–1614. 8784137 10.1161/01.str.27.9.1609

[32] Ma YP, Ma MM, Ge S, Guo RB, Zhang HJ, et al (2007) Intranasally delivered TGF-beta1 enters brain and regulates gene expressions of its receptors in rats. Brain Res Bull 74: 271–277. 10.1016/j.brainresbull.2007.06.021 17720549

[33] Le Y, Yu X, Ruan L, Wang O, Qi D, et al (2005) The immunopharmacological properties of transforming growth factor beta. Int Immunopharmacol 5: 1771–1782. 10.1016/j.intimp.2005.07.006 16275614

[34] Tesseur I, Zou K, Esposito L, Bard F, Berber E, et al (2006) Deficiency in neuronal TGF-beta signaling promotes neurodegeneration and Alzheimer’s pathology. J Clin Invest 116: 3060–3069. 10.1172/JCI27341 17080199 PMC1626127

[35] Bosco P, Ferri R, Salluzzo MG, Castellano S, Signorelli M, et al (2013) Role of the Transforming-Growth-Factor-β1 Gene in Late-Onset Alzheimer’s Disease: Implications for the Treatment. Curr Genomics 14: 147–156. 10.2174/1389202911314020007 24082824 PMC3637679

[36] Caraci F, Spampinato S, Sortino MA, Bosco P, Battaglia G, et al (2012) Dysfunction of TGF-beta1 signaling in Alzheimer’s disease: perspectives for neuroprotection. Cell Tissue Res 347: 291–301. 10.1007/s00441-011-1230-6 21879289

[37] Block ML, Zecca L, Hong JS (2007) Microglia-mediated neurotoxicity: uncovering the molecular mechanisms. Nat Rev Neurosci 8: 57–69. 10.1038/nrn2038 17180163

[38] Meraz-Ríos MA, Toral-Rios D, Franco-Bocanegra D, Villeda-Hernández J, Campos-Peña V (2013) Inflammatory process in Alzheimer’s Disease. Front Integr Neurosci 7: 59 10.3389/fnint.2013.00059 23964211 PMC3741576

[39] Tuppo EE, Arias HR (2005) The role of inflammation in Alzheimer’s disease. Int J Biochem Cell Biol 37: 289–305. 10.1016/j.biocel.2004.07.009 15474976

[40] Heneka MT, O’Banion MK (2007) Inflammatory processes in Alzheimer’s disease. J Neuroimmunol 184: 69–91. 10.1016/j.jneuroim.2006.11.017 17222916

[41] Biscaro B, Lindvall O, Tesco G, Ekdahl CT, Nitsch RM (2012) Inhibition of microglial activation protects hippocampal neurogenesis and improves cognitive deficits in a transgenic mouse model for Alzheimer’s disease. Neurodegener Dis 9: 187–198. 10.1159/000330363 22584394 PMC7068786

[42] Lombardi VR, García M, Rey L, Cacabelos R (1999) Characterization of cytokine production, screening of lymphocyte subset patterns and in vitro apoptosis in healthy and Alzheimer’s Disease (AD) individuals. J Neuroimmunol 97: 163–171. 10.1016/S0165-5728(99)00046-6 10408971

[43] Schindowski K, Eckert A, Peters J, Gorriz C, Schramm U, et al (2007) Increased T-cell reactivity and elevated levels of CD8+ memory T-cells in Alzheimer’s disease-patients and T-cell hyporeactivity in an Alzheimer’s disease-mouse model: implications for immunotherapy. Neuromolecular Med 9: 340–354. 10.1007/s12017-007-8015-9 17963048

[44] Subramanian S, Ayala P, Wadsworth TL, Harris CJ, Vandenbark AA, et al (2010) CCR6: a biomarker for Alzheimer’s-like disease in a triple transgenic mouse model. J Alzheimers Dis 22: 619–629. 10.3233/JAD-2010-100852 20847401 PMC2988888

[45] Lynch MA (2014) The impact of neuroimmune changes on development of amyloid pathology; relevance to Alzheimer’s disease. Immunology 141: 292–301. 10.1111/imm.12156 23876085 PMC3930368

[46] Togo T, Akiyama H, Iseki E, Kondo H, Ikeda K, et al (2002) Occurrence of T cells in the brain of Alzheimer’s disease and other neurological diseases. J Neuroimmunol 124: 83–92. 10.1016/S0165-5728(01)00496-9 11958825

[47] Browne TC, McQuillan K, McManus RM, O’Reilly JA, Mills KH, et al (2013) IFN-γ Production by amyloid β-specific Th1 cells promotes microglial activation and increases plaque burden in a mouse model of Alzheimer’s disease. J Immunol 190: 2241–2251. 10.4049/jimmunol.1200947 23365075

[48] Heath VL, Murphy EE, Crain C, Tomlinson MG, O’Garra A (2000) TGF-beta1 down-regulates Th2 development and results in decreased IL-4-induced STAT6 activation and GATA-3 expression. Eur J Immunol 30: 2639–2649. 10.1002/1521-4141(200009)30:9<2639::AID-IMMU2639>3.0.CO;2-7 11009098

[49] Li M, Shang DS, Zhao WD, Tian L, Li B, et al (2009) Amyloid beta interaction with receptor for advanced glycation end products up-regulates brain endothelial CCR5 expression and promotes T cells crossing the blood-brain barrier. J Immunol 182: 5778–5788. 10.4049/jimmunol.0803013 19380826

[50] Liu YJ, Guo DW, Tian L, Shang DS, Zhao WD, et al (2010) Peripheral T cells derived from Alzheimer’s disease patients overexpress CXCR2 contributing to its transendothelial migration, which is microglial TNF-alpha-dependent. Neurobiol Aging 31: 175–188. 10.1016/j.neurobiolaging.2008.03.024 18462836

[51] Yang YM, Shang DS, Zhao WD, Fang WG, Chen YH (2013) Microglial TNF-α-dependent elevation of MHC class I expression on brain endothelium induced by amyloid-beta promotes T cell transendothelial migration. Neurochem Res 38: 2295–2304. 10.1007/s11064-013-1138-5 23990225

[52] Carare RO, Hawkes CA, Weller RO (2014) Afferent and efferent immunological pathways of the brain. Anatomy, Function and Failure. Brain Behav Immun 36: 9–14. 10.1016/j.bbi.2013.10.012 24145049

[53] Metcalfe MJ, Figueiredo-Pereira ME (2010) Relationship between tau pathology and neuroinflammation in Alzheimer’s disease. Mt Sinai J Med 77: 50–58. 10.1002/msj.20163 20101714 PMC2904237

